# Anxious Traits Intensify the Impact of Depressive Symptoms on Stigma in People Living with HIV

**DOI:** 10.3390/brainsci15080786

**Published:** 2025-07-24

**Authors:** Alexia Koukopoulos, Antonio Maria D’Onofrio, Alessio Simonetti, Delfina Janiri, Flavio Cherubini, Paolo Vassalini, Letizia Santinelli, Gabriella D’Ettorre, Gabriele Sani, Giovanni Camardese

**Affiliations:** 1 Department of Life Science, Health, and Health Professions, Link Campus University, 00165 Rome, Italy; 2 Centro Lucio Bini, Via Crescenzio 42, 00193 Rome, Italy; 3 Department of Neuroscience, Section of Psychiatry, Università Cattolica del Sacro Cuore, 00168 Rome, Italy; 4 Department of Neuroscience, Section of Psychiatry, Fondazione Policlinico Universitario Agostino Gemelli IRCCS, 00168 Rome, Italy; 5 Menninger Department of Psychiatry and Behavioral Sciences, Baylor College of Medicine, Houston, TX 77030, USA; 6 Psychiatry Unit, Faculty of Medicine and Psychology, Psychiatry Residency Training Program, Sapienza University of Rome, 00185 Rome, Italy; 7 Infectious Disease Unit, Sant’Andrea Hospital, Sapienza University of Rome, 00189 Rome, Italy; 8 Department of Public Health and Infectious Diseases, Sapienza University of Rome, 00185 Rome, Italy

**Keywords:** HIV-related stigma, depressive symptoms, affective temperament, internalized stigma, anxious temperament, mental health, psychosocial factors, PLWH, psychometric assessment, moderation analysis

## Abstract

**Background/Objectives**: Despite medical advances, stigma remains a major challenge for people living with HIV (PLWH). This study examined clinical, sociodemographic, and psychological predictors of HIV-related stigma, and explored whether affective temperament moderates the impact of depression on stigma. **Methods**: This cross-sectional observational study included 97 PLWH attending a tertiary infectious disease unit in Rome, Italy. Participants completed a battery of validated psychometric instruments assessing depressive symptoms, anxiety, manic symptoms, mixed affective states, general psychopathology, impulsivity, and affective temperament. HIV-related stigma was evaluated using the Berger HIV Stigma Scale, which measures personalized stigma, disclosure concerns, negative self-image, and concerns with public attitudes. Descriptive statistics were used to characterize the sample. Univariate linear regressions were conducted to explore associations between clinical, psychometric, and sociodemographic variables and each stigma subdimension, as well as the total stigma score. Variables significant at *p* < 0.05 were included in five multivariate linear regression models. Moderation analyses were subsequently performed to assess whether affective temperaments moderated the relationship between significant psychopathological predictors and stigma. Bonferroni correction was applied where appropriate. **Results**: Higher depressive symptom scores are significantly associated with greater internalized stigma (B = 0.902, *p* = 0.006) and total stigma (B = 2.603, *p* = 0.008). Furthermore, moderation analyses showed that anxious temperament significantly intensified the relationship between depressive symptoms and both negative self-image (interaction term B = 0.125, *p* = 0.001) and total stigma (B = 0.336, *p* = 0.002). **Conclusions**: Depressive symptoms and anxious temperament are associated with HIV-related stigma. Integrating psychological screening and targeted interventions for mood and temperament vulnerabilities may help reduce stigma burden in PLWH and improve psychosocial outcomes.

## 1. Introduction

Human immunodeficiency virus (HIV) remains a global public health concern [1] not only due to its biological impact but also due to the persistent stigma faced by people living with HIV (PLWH) [2]. Despite significant medical progress in the treatment and management of HIV, stigma continues to exert a profound influence on the psychological well-being and quality of life of affected individuals [2]. HIV-related stigma is a multifaceted construct encompassing enacted, anticipated, and internalized dimensions [3], and has been associated with a range of negative outcomes including reduced medication adherence [4], disengagement from healthcare services [5], impaired social functioning [6], and increased psychiatric morbidity [7,8].

Psychiatric comorbidity is particularly prevalent among PLWH [9], who experience disproportionately high rates of depression [10], anxiety [11], substance use disorders [12], and trauma-related syndromes [13]. These conditions often emerge in the context of complex psychosocial stressors, such as marginalization [14], chronic illness burden [15], and anticipated discrimination [16]. In response to the unique mental health needs of this population, a dedicated subspecialty, HIV psychiatry, has emerged at the intersection of infectious disease and mental health. This field integrates psychotherapeutic, psychopharmacological, and psychosocial approaches, addressing both syndromal psychopathology and broader challenges such as stigma, identity, and treatment engagement [17,18]. Given the high prevalence and potential impact of these comorbidities on HIV outcomes, early and systematic mental health screening is essential in the context of HIV care, allowing for timely identification and intervention [19].

Although previous studies have investigated the association between psychopathological symptoms and perceived HIV-related stigma, the vast majority have focused on how stigma may exacerbate or contribute to the onset of psychological distress among PLWH [20,21]. In contrast, the present study addresses this relationship from the opposite direction, seeking to understand how psychopathological symptoms may influence the perception of stigma. To date, no research has systematically examined how state-dependent clinical dimensions and trait-level affective dispositions may interact in shaping stigma experiences in this population.

Affective temperament refers to stable biologically based dispositions that influence an individual’s emotional reactivity and baseline mood tone. As conceptualized by Akiskal and colleagues [22,23,24], affective temperaments (e.g., depressive, cyclothymic, hyperthymic, irritable, and anxious) represent subclinical affective styles that can predispose individuals to specific psychiatric syndromes. A growing body of evidence has demonstrated that certain temperamental profiles, such as cyclothymic and anxious temperaments, are associated with increased vulnerability to mood disorders, affective instability, and poor psychosocial functioning [25,26,27,28,29]. These temperaments not only influence the onset and course of psychiatric symptoms but also shape how individuals interpret and respond to stressful interpersonal and social stimuli, including stigmatizing experiences [30,31]. However, it is important to acknowledge that measuring affective temperament may be subject to certain limitations, particularly in distinguishing enduring temperamental traits from current clinical symptoms, which may transiently influence self-report measures [32,33].

Building on this gap, we adopt a theoretically driven approach, informed by models of self-stigma (e.g., Corrigan’s progressive model of self-stigma in depression, which outlines successive stages of stereotype awareness → personal agreement → self-concurrence → harm to self) [34,35] and psychological vulnerability (drawing on cognitive vulnerability–stress and diathesis–stress frameworks, which conceptualize psychopathology as arising from the interaction between stable predispositions and environmental stressors) [36,37] to explore the combined impact of current psychopathological burden and affective temperament on perceived HIV-related stigma. By integrating both state (e.g., depression, anxiety, mixed states, impulsivity, general psychopathology) and trait (temperamental) dimensions through a rigorous and comprehensive assessment, our study aims to characterize the psychological mechanisms underlying stigma perception and clarify the directionality of this relationship, which remains underexplored in the literature.

We analyzed data from a well-characterized cohort of PLWH receiving care in a specialized infectious disease unit who underwent a comprehensive and standardized psychopathological assessment. This included key symptomatic dimensions such as depression, anxiety, mixed states, manic symptoms, impulsivity, and global psychopathological distress, as well as affective temperament traits. By integrating these dimensions of state and trait, our aim was to identify the psychological profiles most strongly associated with stigma perception.

The primary objective of this study was to identify clinical, sociodemographic, and psychometric predictors of the different dimensions of HIV-related stigma, specifically enacted, anticipated, and internalized stigma. A secondary aim was to test whether affective temperament moderates the association between psychopathological symptoms and perceived stigma. This approach offers a new understanding of the individual-level determinants of stigma and may inform the design of more effective targeted psychosocial interventions for PLWH.

## 2. Materials and Methods

This study is part of the PSYCH+ project, a single-center, cross-sectional, observational, and non-profit study conducted in Italy by the Department of Infectious Diseases of the Policlinico Umberto I Hospital in Rome. The coordinating center is the University of Rome “Sapienza”.

### 2.1. Participants

Patients were enrolled in a naturalistic and consecutive manner based on their routine access to outpatient care (from August 2024 to March 2025) and had provided informed consent to participate. Inclusion criteria were as follows: (1) the ability to understand and sign valid informed consent; (2) age between 18 and 65 years; (3) confirmed diagnosis of HIV infection; and (4) Mini-Mental State Examination (MMSE) score ≥ 24. Exclusion criteria included (1) any current unstabilized psychiatric disorder (e.g., major depression, psychotic disorders), (2) acute substance intoxication within the past month, (3) major central nervous system opportunistic infection in the past 12 months (e.g., cryptococcal meningitis), (4) pregnancy, and (5) inadequate cognitive or language capacity (e.g., MMSE < 24 or non-Italian speaker) to ensure clear and valid participation.

### 2.2. Data Collection

Demographic, clinical, and therapeutic data were obtained from medical records. Psychiatric assessment was conducted during clinical interviews using both clinician-administered and self-administered instruments.

Clinician-administered instruments included the SCID-5-CV (Structured Clinical Interview for DSM-5, clinical version) [38] and the SCID-5-PD (Structured Clinical Interview for DSM-5, personality disorders) [39] to assess current and lifetime psychiatric and personality disorders, respectively. Depressive symptoms were evaluated using the Hamilton Depression Rating Scale (HAMD), a 17-item scale with four additional items addressing diurnal variation and atypical features such as depersonalization, paranoid ideation, and obsessive–compulsive symptoms [40]. Anxiety was measured through the Hamilton Anxiety Rating Scale (HAMA), which includes 14 items capturing both psychic and somatic symptoms [41]. Manic symptoms were assessed with the Young Mania Rating Scale (YMRS), an 11-item instrument that evaluates the severity of manic episodes [42]. The Koukopoulos Mixed Depression Rating Scale (KMDRS), consisting of 14 items, was used to assess mixed features in patients with a history of major depressive episodes [43]. General psychopathology was assessed using the Brief Psychiatric Rating Scale (BPRS), an 18-item instrument that evaluates a wide range of psychiatric symptoms including psychosis, anxiety, and mood disturbances [44]. Global cognitive functioning was screened using the MMSE [45]. All clinician-administered instruments were conducted by trained psychiatrists. To ensure consistency and minimize bias, cognitive screening (MMSE) was conducted first, followed by the clinician-administered interviews and rating scales. Subsequently, participants completed the self-report questionnaires in a quiet and private setting. When necessary, any items perceived as unclear or difficult by the participants were clarified by a research assistant without influencing their responses. All instruments used in this study were administered using their validated Italian versions.

Self-administered instruments included the Symptom Checklist-90 Revised (SCL-90-R), a 90-item questionnaire assessing symptomatology across 9 domains—somatization, obsessive–compulsive symptoms, depression, anxiety, hostility, phobic anxiety, paranoid ideation, psychoticism, and interpersonal sensitivity—rated on a 5-point Likert scale [46]. The Barratt Impulsiveness Scale (BIS-11) was employed to assess impulsivity across six first-order dimensions (attention, motor impulsiveness, self-control, cognitive complexity, perseverance, cognitive instability) and three second-order factors (attentional, motor, and non-planning impulsivity) [47]. Affective temperament traits were evaluated with the Temperament Evaluation of Memphis, Pisa, Paris and San Diego (TEMPS-A), a 110-item true/false questionnaire that identifies depressive, cyclothymic, hyperthymic, irritable, and anxious temperamental profiles [24]. The Berger HIV Stigma Scale was used to assess perceived stigma associated with HIV through 40 items covering enacted, anticipated, and internalized stigma domains, with total scores ranging from 40 to 160 [48].

### 2.3. Statistical Analysis

Descriptive statistics were computed to characterize the sociodemographic and clinical features of the sample. Univariate linear regression analyses were initially performed to examine the associations between individual clinical, psychometric, and sociodemographic variables and each of the four HIV-related stigma subdimensions (personalized stigma, disclosure concerns, negative self-image, and public attitudes), as well as the total stigma score. Variables that showed a significant association at *p* < 0.05 in these univariate analyses were retained for inclusion in the subsequent multivariate models. Five separate multivariate linear regression (MLR) analyses were then conducted, each corresponding to one stigma subdimension or the total stigma score as the dependent variable. This approach allowed for a more precise estimation of independent associations while accounting for potential confounding among predictors. Building on the results of the multiple linear regression analyses, moderation analyses were subsequently conducted to examine whether affective temperaments (as assessed by the TEMPS-A subscales) moderated the relationship between the psychopathological dimension that had emerged as a significant predictor of the stigma-related dimensions. When appropriate, Bonferroni correction was applied in the regression models to control for type I error due to multiple comparisons. All statistical analyses were performed using IBM SPSS Statistics (version 26), and significance was set at the corrected alpha level for each analytic step.

**Figure 1:**
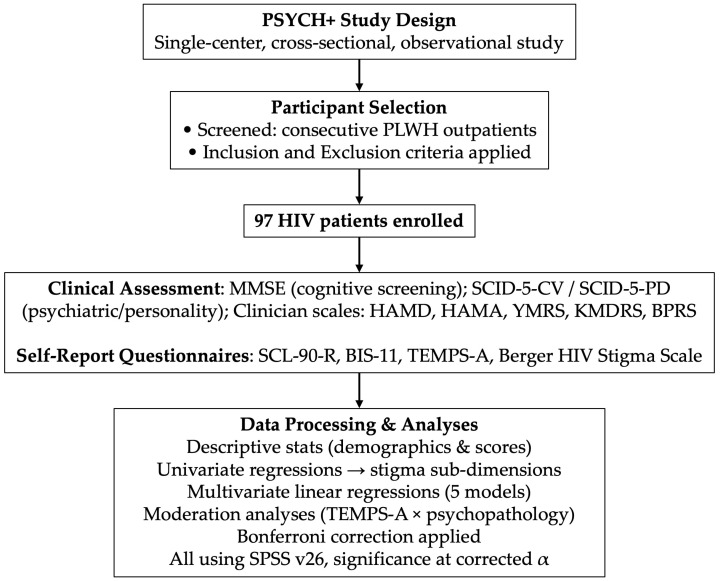
Flowchart of the PSYCH+ study design and data processing workflow. Note: BIS-11 (Barratt Impulsiveness Scale-11), BPRS (Brief Psychiatric Rating Scale), HAMA (Hamilton Anxiety Rating Scale), HAMD (Hamilton Depression Rating Scale), KMDRS (Koukopoulos Mixed Depression Rating Scale), MMSE (Mini-Mental State Examination), SCID-5-CV (Structured Clinical Interview for DSM-5, clinical version), SCID-5-PD (Structured Clinical Interview for DSM-5, personality disorders version), SCL-90-R (Symptom Checklist-90-Revised), TEMPS-A (Temperament Evaluation of Memphis, Pisa, Paris and San Diego—short Italian version), YMRS (Young Mania Rating Scale).

A block diagram summarizing the study design and methods of data processing can be found in Figure 1.

## 3. Results

### 3.1. Descriptive Analysis

A total of 97 patients living with HIV were enrolled in this study. The sociodemographic and clinical characteristics of the sample are detailed in Table 1.

### 3.2. Regression Analysis

#### 3.2.1. Univariate Regression Analysis

We conducted several univariate linear regression analyses using psychometric (HAMD, HAMA, YMRS, KMDRS, BPRS, SCL-90, BIS), clinical (CD4+ levels, years since diagnosis, years of ART therapy, number of ART medications taken, current psychiatric disorder, past psychiatric disorder, past psychiatric hospitalization, past suicide attempts, family history of psychiatric disorders, number of psychotropic drugs taken, current substance use, past substance use), and sociodemographic (age, gender, marital status, level of education, employment status) variables as predictors of the different dimensions of HIV-related stigma (personalized stigma, disclosure concerns, negative self-image, concerns with public attitudes, total stigma score). Details of the univariate linear regression models, including coefficients, significance levels, and confidence intervals, are reported in Supplementary Table S1.

Several psychological variables emerged as significant predictors of personalized stigma in univariate regression analyses. Higher depressive symptoms (HAMD: β = 0.431, *p* < 0.001) and anxiety symptoms (HAMA: β = 0.371, *p* < 0.001) were strongly associated with greater stigma. General psychopathology, as assessed by the BPRS (β = 0.328, *p* = 0.001), and mixed affective features measured by the KMDRS (β = 0.227, *p* = 0.030), were also significantly associated. Multiple SCL-90-R subscales showed positive associations with personalized stigma. In particular, somatization (β = 0.233, *p* = 0.023), obsessive–compulsive symptoms (β = 0.340, *p* = 0.001), interpersonal sensitivity (β = 0.398, *p* < 0.001), depression (β = 0.336, *p* = 0.001), anxiety (β = 0.387, *p* < 0.001), hostility (β = 0.305, *p* = 0.003), phobic anxiety (β = 0.321, *p* = 0.002), paranoid ideation (β = 0.283, *p* = 0.005), and psychoticism (β = 0.389, *p* < 0.001) were all significantly related. In addition, higher scores on the Global Severity Index (GSI: β = 0.372, *p* < 0.001), the positive symptom total (PST: β = 0.370, *p* < 0.001), and the Positive Symptom Distress Index (PSDI: β = 0.341, *p* = 0.001) were associated with higher personalized stigma. Among impulsivity traits, both attentional impulsiveness (BIS A: β = 0.263, *p* = 0.010) and attentional instability (BIS AI: β = 0.230, *p* = 0.027) were significantly associated with greater personalized stigma.

Several psychological variables were significantly associated with disclosure concerns. Depressive symptoms (HAMD: β = 0.350, *p* = 0.001) and anxiety symptoms (HAMA: β = 0.285, *p* = 0.005) predicted greater stigma related to disclosure. General psychiatric distress, as measured by the BPRS (β = 0.321, *p* = 0.002), was also significantly associated with disclosure concerns. Multiple SCL-90-R subscales showed the following significant positive associations: somatization (β = 0.253, *p* = 0.013), obsessive–compulsive symptoms (β = 0.298, *p* = 0.003), interpersonal sensitivity (β = 0.421, *p* < 0.001), depression (β = 0.338, *p* = 0.001), anxiety (β = 0.352, *p* < 0.001), hostility (β = 0.358, *p* < 0.001), phobic anxiety (β = 0.254, *p* = 0.013), paranoid ideation (β = 0.248, *p* = 0.016), psychoticism (β = 0.387, *p* < 0.001), Global Severity Index (GSI: β = 0.358, *p* < 0.001), positive symptom total (PST: β = 0.363, *p* < 0.001), and Positive Symptom Distress Index (PSDI: β = 0.383, *p* < 0.001). Among impulsivity traits, only attentional impulsiveness (BIS A: β = 0.206, *p* = 0.045) showed a significant association. In terms of sociodemographic and clinical variables, gender (β = 0.236, *p* = 0.020), unemployment (β = −0.297, *p* = 0.003), and current psychiatric disorder (β = 0.205, *p* = 0.046) were also significant predictors of disclosure concerns.

Negative self-image was significantly associated with numerous psychological variables. Depressive (HAMD: β = 0.506, *p* < 0.001) and anxiety (HAMA: β = 0.418, *p* < 0.001) symptoms were robust predictors. Both general psychopathology (BPRS: β = 0.362, *p* < 0.001) and mixed affective states (KMDRS: β = 0.264, *p* = 0.011) were also significantly associated. Among SCL-90-R subscales, the following were significantly related to higher negative self-image: somatization (β = 0.324, *p* < 0.001), obsessive–compulsive (β = 0.395, *p* < 0.001), interpersonal sensitivity (β = 0.470, *p* < 0.001), depression (β = 0.412, *p* < 0.001), anxiety (β = 0.453, *p* < 0.001), hostility (β = 0.418, *p* < 0.001), phobic anxiety (β = 0.315, *p* = 0.002), paranoid ideation (β = 0.314, *p* = 0.002), psychoticism (β = 0.475, *p* < 0.001), Global Severity Index (GSI: β = 0.450, *p* < 0.001), positive symptom total (PST: β = 0.407, *p* < 0.001), and Positive Symptom Distress Index (PSDI: β = 0.461, *p* < 0.001). Regarding impulsivity, both attentional impulsiveness (BIS A: β = 0.231, *p* = 0.025) and attentional instability (BIS AI: β = 0.216, *p* = 0.032) subscales were significantly associated with higher negative self-image. Among sociodemographic and clinical variables, lower level of education (β = −0.210, *p* = 0.017), unemployment (β = −0.270, *p* = 0.009), and family history of psychiatric disorders (β = 0.206, *p* = 0.044) were also significant predictors.

Perceptions of negative public attitudes toward PLWHA were significantly associated with psychological distress. Higher HAMD (β = 0.429, *p* < 0.001) and HAMA (β = 0.376, *p* < 0.001) scores, as well as BPRS (β = 0.307, *p* < 0.001) and KMDRS (β = 0.212, *p* = 0.043), were all significantly associated. SCL-90-R subscales significantly associated with this domain included the following: obsessive–compulsive (β = 0.340, *p* = 0.001), interpersonal sensitivity (β = 0.409, *p* < 0.001), depression (β = 0.357, *p* < 0.001), anxiety (β = 0.369, *p* < 0.001), hostility (β = 0.353, *p* < 0.001), phobic anxiety (β = 0.314, *p* = 0.002), paranoid ideation (β = 0.288, *p* = 0.005), psychoticism (β = 0.400, *p* < 0.001), Global Severity Index (β = 0.369, *p* < 0.001), positive symptom total (β = 0.342, *p* = 0.001), and Positive Symptom Distress Index (β = 0.385, *p* < 0.001). Regarding impulsivity traits, both the attentional impulsiveness (BIS A: β = 0.280, *p* = 0.006) and attentional instability (BIS AI: β = 0.214, *p* = 0.035) subscales were significantly associated with more negative perceptions of public attitudes toward PLWHA.

Total stigma score was significantly predicted by depressive (HAMD: β = 0.464, *p* < 0.001) and anxiety (HAMA: β = 0.392, *p* < 0.001) symptoms, as well as global psychopathology (BPRS: β = 0.365, *p* < 0.001) and mixed affective states (KMDRS: β = 0.217, *p* = 0.037). SCL-90-R subscales significantly associated with the total score included the following: somatization (β = 0.259, *p* = 0.011), obsessive–compulsive (β = 0.340, *p* = 0.001), interpersonal sensitivity (β = 0.452, *p* < 0.001), depression (β = 0.373, *p* < 0.001), anxiety (β = 0.408, *p* < 0.001), hostility (β = 0.376, *p* < 0.001), phobic anxiety (β = 0.299, *p* = 0.001), paranoid ideation (β = 0.298, *p* = 0.003), psychoticism (β = 0.432, *p* < 0.001), Global Severity Index (β = 0.404, *p* < 0.001), positive symptom total (β = 0.388, *p* < 0.001), and Positive Symptom Distress Index (β = 0.412, *p* < 0.001). Attentional impulsiveness (BIS A: β = 0.261, *p* = 0.011) and attentional instability (BIS AI: β = 0.220, *p* = 0.027) were the only impulsivity domains associated with the total stigma score. In terms of sociodemographic variables, unemployment was also a predictor (β = −0.244, *p* = 0.018).

#### 3.2.2. Multivariate Regression Analysis

To account for potential confounding factors, five separate multivariate linear regression (MLR) analyses were conducted—one for each of the four HIV-related stigma subdimensions (personalized stigma, disclosure concerns, negative self-image, and concerns with public attitudes), as well as the total stigma score. Predictors included only those variables that showed significant associations in the univariate analyses, encompassing selected psychometric, clinical, and sociodemographic factors. Prior to interpreting the results of the five multivariate linear regression models, standard assumptions were systematically evaluated. Normality of residuals was assessed through the visual inspection of histograms and normal P–P plots, while homoscedasticity was evaluated using scatterplots of standardized residuals versus predicted values. The Durbin–Watson statistic indicated the acceptable independence of residuals across all models (values ranged from 1.990 to 2.240). Residuals were approximately normally distributed, and no major violations of homoscedasticity were observed. However, variance inflation factor (VIF) values revealed substantial multicollinearity, particularly among SCL-90 subscales, with several exceeding conventional thresholds (e.g., VIF > 10) and extreme values observed for the GSI (VIF > 700 in some models). This finding was expected given the inherent intercorrelation among SCL-90 dimensions, which are conceptually and psychometrically related. Details of the five multivariate linear regression models, including coefficients, significance levels, and confidence intervals, are reported in Supplementary Table S2.

The first MLR was conducted with personalized stigma as the dependent variable. None of the included predictors reached statistical significance at *p* < 0.05, indicating that no psychological or clinical variables independently predicted levels of personalized stigma in the adjusted model.

The second MLR was conducted with disclosure concerns as the dependent variable. Higher scores on SCL-90-R INT (B = 8.055, *p* = 0.013) and being unemployed (B = −3.311, *p* = 0.040) were significantly associated with greater disclosure-related stigma.

The third MLR was conducted with negative self-image as the dependent variable. Higher HAMD scores (B = 0.902, *p* = 0.006) and being unemployed (B = −3.931, *p* = 0.048) were significantly associated with greater internalized stigma related to self-image.

The fourth MLR was conducted with public attitudes about PLWHA as the dependent variable. Higher scores on HAMD (B = 1.088, *p* = 0.041) and SCL-90-R INT (B = 11.566, *p* = 0.012) were significantly associated with greater perceived public stigma. Conversely, higher SCL-90-R GSI scores were associated with lower perceived stigma (B = −51.107, *p* = 0.033).

The fifth MLR was conducted with the total stigma score as the dependent variable. Higher HAMD scores (B = 2.603, *p* = 0.008) and higher SCL-90-R INT scores (B = 27.537, *p* = 0.017) were significantly associated with greater overall perceived stigma.

After applying the Bonferroni correction to account for multiple comparisons (α_adjusted_ = 0.05/5 = 0.01), only HAMD scores in the third and fifth multivariate regression models remained statistically significant predictors of stigma outcomes. This correction was applied given that five separate regressions were performed, and the dependent variables (stigma subscales) were highly correlated.

#### 3.2.3. Moderation Analysis

Moderation analyses were performed to investigate whether affective temperament traits modified the association between depressive symptoms and perceived HIV-related stigma. Two separate multivariate linear regression models were conducted using the interaction terms between HAMD scores and each TEMPS-A subscale (dysthymic, cyclothymic, hyperthymic, irritable, and anxious) as predictors. The dependent variables were the negative self-image subdimension and the total stigma score, respectively.

In the model with negative self-image as the dependent variable, a significant interaction was observed between anxious temperament and depressive symptoms (B = 0.125, *p* = 0.001), as well as between hyperthymic temperament and depressive symptoms (B = 0.088, *p* = 0.027). In the model with total stigma score as the outcome, the anxious temperament × HAMD interaction remained significant (B = 0.336, *p* = 0.002).

To account for multiple testing, a Bonferroni correction was applied (α_adjusted_ = 0.05/2 = 0.025). After correction, only the interaction between anxious temperament and depressive symptoms remained a statistically significant predictor of both negative self-image (B = 0.125, *p* = 0.001) and total stigma score (B = 0.336, *p* = 0.002), suggesting that anxious temperament may amplify the impact of depressive symptoms on internalized and overall stigma.

**Figure 2:**
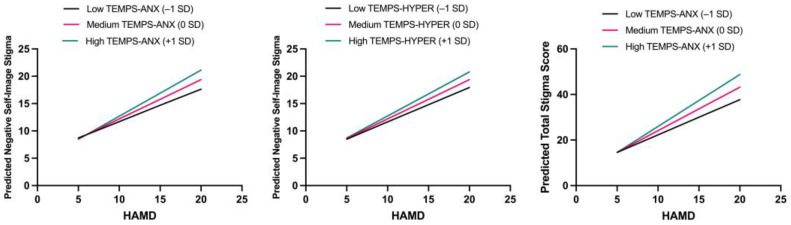
Moderating effects of affective temperaments on the relationship between depressive symptoms (HAMD) and HIV-related stigma. (**Left**): Interaction between anxious temperament and depressive symptoms in predicting negative self-image. (**Center**): Interaction between hyperthymic temperament and depressive symptoms in predicting negative self-image. (**Right**): Interaction between anxious temperament and depressive symptoms in predicting total stigma score. Stigma scores are plotted at low (−1 SD), medium (mean), and high (+1 SD) levels of the corresponding temperament traits.

To aid interpretation, the interaction effects are illustrated in Figure 2. Detailed results are reported in Table 2.

## 4. Discussion

This study aimed to identify the key determinants of HIV-related stigma, with a particular emphasis on psychopathological factors. Through the analysis of a comprehensive set of clinical, sociodemographic, and psychometric variables, our findings underscore the prominent role of depressive symptoms in shaping stigma experiences among individuals living with HIV. Specifically, depression was independently associated with greater internalized stigma, particularly negative self-image, as well as higher overall stigma levels, suggesting that depressive symptomatology is a robust and consistent correlation of perceived stigma in this population. A distinctive contribution of this study lies in its theoretical and methodological perspective, i.e., rather than treating stigma as a cause of psychological distress—as is frequently the case in the literature—we investigated how psychopathological vulnerability may be associated with the development and perception of stigma. In addition, by considering both state-dependent factors (e.g., current depressive symptoms) and stable individual traits (e.g., affective temperament), the analysis provides a comprehensive understanding of the mechanisms that could be associated with higher stigma sensitivity. Notably, anxious temperament significantly amplified the relationship between depressive symptoms and perceived stigma, suggesting that individuals with elevated trait anxiety experience stigma more intensely.

Building upon our findings, the robust association between depressive symptoms and HIV-related stigma has been consistently supported by a growing body of the literature. However, most prior research has conceptualized stigma as a contributing factor to psychological distress. A systematic review by MacLean and Wetherall (2021) [21] concluded that all forms of stigma were significantly associated with depressive symptoms among PLWH, particularly in South African populations. Notably, some longitudinal studies included in the review suggested a bidirectional relationship, wherein stigma predicted later depression in one study, and depression predicted future stigma in another, highlighting the dynamic interaction between these constructs. Similarly, a recent meta-analysis by Desta et al. (2024) [49] confirmed a strong overall association between perceived stigma and depression, reinforcing the importance of integrating mental health screenings and targeted interventions into HIV care programs. This relationship has also been extensively explored in longitudinal designs. For instance, Yuan et al. (2023) [50], using a four-wave panel study, demonstrated that depressive symptoms not only followed but also preceded increases in both internalized and anticipated stigma, suggesting reciprocal reinforcement over time. They further clarified these dynamics by showing that social support and resilience may mediate the longitudinal path from internalized stigma to depression and vice versa [51]. These findings suggest that depression and stigma do not operate in isolation but rather interact within a broader psychosocial context that includes individual vulnerabilities and protective resources.

In our study, we observed a particularly strong association between depressive symptoms and the negative self-image subdimension of HIV-related stigma. This subscale captures deeply internalized and self-directed aspects of stigma, including feelings of guilt, shame, and personal devaluation due to HIV status. Statements such as “I feel guilty because I have HIV”, “I feel I am not as good a person as others because I have HIV”, “Having HIV makes me feel unclean”, “Having HIV makes me feel I’m a bad person”, and “Having HIV in my body is disgusting to me” directly reflect cognitive and emotional patterns frequently observed in depression. These items strongly mirror core depressive features such as self-blame [52], guilt [53], feelings of worthlessness [54], and disgust directed toward the self [55]. The pervasive tone of guilt, inferiority, and moral self-judgment embedded in these statements is emblematic of the distorted self-referential thinking that characterizes depressive psychopathology [56]. This conceptual overlap may partially explain the strong association observed in our sample. However, it is important to note that these mechanisms are complex and likely bidirectional. The link between depressive symptoms and internalized stigma may also be mediated by broader psychosocial factors, such as social rejection [57,58], emotion regulation [59], or a history of trauma [60]. Therefore, the association observed may not reflect a direct causal relationship but rather a shared psychological dimension rooted in self-referential negative thinking.

To the best of our knowledge, this is the first study to assess not only psychopathological correlates but also the moderating role of affective temperament in the stigma–depression link among people living with HIV. Anxious temperament, as conceptualized by the TEMPS-A framework, is characterized by pervasive worry, anticipatory anxiety, heightened sensitivity to social evaluation, and a tendency to ruminate on potential threats or negative outcomes [61]. These enduring traits may predispose individuals to perceive greater social rejection or internalize stigmatizing messages more readily. Individuals with anxious temperamental traits often exhibit cognitive patterns such as catastrophizing, hypervigilance to threat, and self-focused attention [62,63], all of which may exacerbate depressive cognitions and emotions in the context of stigmatization. In this sense, anxious temperament may function as a psychological vulnerability that amplifies the emotional burden of stigma, especially when compounded by concurrent depressive symptoms.

## 5. Limitations

Despite its strengths, this study also has some limitations that should be acknowledged. First, its cross-sectional design precludes any conclusions about causality between depressive symptoms, affective temperament, and stigma. Second, the use of self-report instruments may have introduced reporting biases, particularly in the assessment of sensitive constructs such as stigma and psychopathological symptoms. Third, the sample size of ninety-seven participants is relatively limited, which may reduce the statistical power of moderation analyses, particularly in detecting interaction effects. Fourth, the sample was derived from a single clinical setting, which may limit the generalizability of the findings to broader and more diverse populations of people living with HIV. In particular, the experience and impact of stigma are likely to vary across different cultural, socioeconomic, and regional contexts, further limiting the external validity of the results. Moreover, given that this study was conducted in Italy, it is important to consider the influence of specific cultural and territorial factors—such as prevailing social norms, public attitudes toward HIV and mental health, and structural aspects of the Italian healthcare system—which may shape both the perception of stigma and the expression of affective temperaments. Fifth, our use of the MMSE (cut-off ≥ 24) restricts the sample to cognitively preserved individuals, which may limit generalizability to PLWH with subtler cognitive impairment. This choice was made because our study focuses on outcomes—such as stigma and temperament—that require sufficient cognitive capacity for valid self-report, and assessing cognitively impaired individuals would require a separate study design. Sixth, an additional limitation concerns the analytical strategy. We performed separate regression analyses for each dimension of HIV-related stigma, which increased the number of statistical tests. Although we applied Bonferroni correction to reduce the risk of type I error, this approach may have reduced the overall statistical power and increased the likelihood of type II errors. Seventh, another limitation is the absence of a priori power analysis. As participants were enrolled in a naturalistic and consecutive manner during routine clinical activity, the final sample size was not determined based on statistical power considerations. While this reflects real-world conditions, it may limit the ability to detect smaller effects, particularly in multivariable analyses. Eight, while this study focused on depressive symptoms and specific affective temperaments (e.g., anxious, hyperthymic), we recognize that a range of other clinical and psychopathological factors—such as generalized anxiety disorders, personality disorders, and other mental health dimensions—may influence the experience of stigma. The variables considered here are not intended to be exhaustive. Ninth, a major limitation of our multivariate models is the presence of multicollinearity among the psychometric predictors, particularly the SCL-90 subscales. Although this was expected given the high conceptual overlap between these dimensions, it may have influenced the stability and interpretability of individual regression coefficients. Future studies may consider variable selection strategies or the use of composite indices (e.g., global severity scores) to mitigate collinearity and enhance model robustness. Lastly, while affective temperament was operationalized using a well-validated tool (TEMPS-A), other personality dimensions and contextual psychosocial variables were not assessed and may also play a role in stigma processes. Moreover, although our findings suggest that anxious temperament amplifies the impact of depressive symptoms on stigma, temperament is a complex and multifaceted construct. The interpretation of its moderating role should be viewed with caution and would benefit from confirmation in longitudinal studies and through more in-depth analysis of individual psychosocial contexts.

## 6. Conclusions

Our findings underscore the prominent role of depressive symptoms in shaping stigma experiences among individuals living with HIV. Depression was independently associated with greater internalized stigma—particularly negative self-image—as well as higher overall stigma levels. We observed a particularly strong association between depressive symptoms and the negative self-image subdimension of HIV-related stigma. Moreover, anxious temperament significantly amplified the relationship between depressive symptoms and perceived stigma.

These findings carry important clinical implications for the care of PLWHA. While the condition itself constitutes a foundational vulnerability, particularly in psychosocial terms, our results emphasize the need to consider the unique psychological and temperamental profiles of each person when designing interventions. Affective traits such as anxious temperament and comorbid depressive symptoms may heighten the internalization of stigma, reinforcing negative self-perceptions and emotional distress. From a clinical perspective, this suggests that beyond treating the biological aspects of HIV, it is essential to integrate tailored psychological support targeting affective vulnerabilities. Interventions such as cognitive behavioral therapy, resilience building, and psychoeducation could mitigate the stigma-related burden, especially in patients displaying heightened anxiety and depressive traits. Furthermore, early screening for mood disturbances and temperament patterns could help identify individuals at greater risk of experiencing stigma-related distress, allowing for timely and targeted support. Importantly, these individual-level interventions must be accompanied by broader societal and policy-level efforts aimed at reducing misinformation, fostering inclusive narratives, and promoting structural changes that combat HIV-related stigma. Only by addressing both internal and external determinants can we hope to alleviate the full spectrum of stigma experienced by PLWHA.

## Figures and Tables

**Table 1:** Characteristics of the sample. The instruments and scoring guidelines are as follows: HAMD (17-item): 0–7 = no depression/remission; 8–13 = mild; 14–18 = moderate; 19–22 = severe; ≥23 = very severe depression. HAMA: 0–13 = no/mild anxiety; 14–17 = moderate; ≥ 18 = severe, with ≥14 often used to define clinically significant anxiety. YMRS: <12 = no clinical mania; 12–20 = mild to moderate; 21–25 = moderate; ≥26 = severe; a score ≥20 is frequently adopted as the clinical threshold. BPRS (18 items): total scores > 30 suggest clinically relevant psychopathology. SCL-90-R: raw scores were used in our analysis; the GSI raw mean (>0.9) is considered clinically significant in medical populations. BIS-11: scores > 70 are indicative of high impulsivity in normative samples. TEMPS-A (Italian 39-item version): cut-offs per temperament subtype are depressive ≥ 13, cyclothymic ≥ 14, hyperthymic ≥ 14, irritable ≥ 13, and anxious ≥ 13. Berger HIV Stigma Scale (40 items): score range 40–160; no standardized clinical cut-off exists, but stigma levels are typically categorized using tertiles or percentiles (e.g., “high stigma” = ≥75th percentile). Please refer to the Materials and Methods section and listed references for detailed justification of cut-off selections. Note: A (attention); AI (attentional impulsiveness); ANX (anxiety/anxious); ART (antiretroviral therapy); BIS (Barratt Impulsiveness Scale); BPRS (Brief Psychiatric Rating Scale); CC (cognitive complexity); CI (cognitive instability); CYCL (cyclothymic); CwPA (concern with public attitudes); DC (disclosure concerns); DEP (depression); DYS (depressive); GSI (Global Severity Index); HAMA (Hamilton Anxiety Rating Scale); HAMD (Hamilton Depression Rating Scale); HOS (hostility); HYPER (hyperthymic); IRR (irritable); I-S (interpersonal sensitivity); KMDRS (Koukopoulos Mixed Depression Rating Scale); M (motor); MI (motor impulsiveness); NPI (non-planning impulsiveness); NSI (negative self-image); O-C (obsessive–compulsive); P (perseverance); PAR (paranoid ideation); PHOB (phobic anxiety); PS (personalized stigma); PSDI (Positive Symptom Distress Index); PST (positive symptom total); PSY (psychoticism); SC (self-control); SCL-90-R (); SOM (somatization); TEMPS (Temperament Evaluation of Memphis, Pisa, Paris and San Diego questionnaire); YMRS (Young Mania Rating Scale).

Variable	Value
**Age**, mean (SD)	51.03 ± 11.32
**Gender**, %	69.1% Male
	28.9% Female
	2.1% Transgender
**Marital status**, %	49.5% Single
	37.1% Married or living with a partner
	9.3% Separated/divorced
	4.1% Widowed
**Level of education**, %	1% Elementary school
	9.3% Middle school
	57.7% High school
	32.2% University degree
**Employment status**, %	23.7% Unemployed
	75.3% Employed
**Years from diagnosis**, mean (SD)	15.75 ± 9.61
**CD4+ levels (cells/mm^3^)**, mean (SD)	633.19 ± 240.05
**Years of ART therapy**, mean (SD)	14.24 ± 8.95
**Number of ART medications taken**, mean (SD)	2.89 ± 0.81
**Past psychiatric disorder**, %	63.5% No
	36.5% Yes
**Current psychiatric disorder**, %	75.0% No
	25.0% Yes
**Past psychiatric hospitalization**, %	97.9% No
	2.1% Yes
**Past suicide attempts**, %	97.9% No
	2.1% Yes
**Past psychopharmacological treatment**, %	67.7% No
	32.3% Yes
**Current psychopharmacological treatment**, %	76.0% No
	24.0% Yes
**Number of psychotropic drugs taken,** mean (SD)	0.34 ± 0.69
**Family history of psychiatric disorders**, %	68.0% No
	32.0% Yes
**Past substance use**, %	71.1% No
	28.9% Yes
**Current substance use**, %	88.7% No
	11.3% Yes
**HAMD**, mean (SD)	6.95 ± 5.69
**HAMA**, mean (SD)	6.47 ± 5.57
**YMRS**, mean (SD)	4.40 ± 4.22
**KMDRS**, mean (SD)	8.10 ± 3.99
**BPRS**, mean (SD)	28.95 ± 7.71
**SCL90-R**, mean (SD)	
**SOM**	0.69 + 0.64
**O-C**	0.80 + 0.79
**I-S**	0.64 + 0.68
**DEP**	0.76 + 0.79
**ANX**	0.62 + 0.62
**HOS**	0.47 + 0.62
**PHOB**	0.21 + 0.38
**PAR**	0.69 + 0.69
**PSY**	0.52 + 0.61
**GSI**	0.64 + 0.59
**PST**	32.25 + 21.68
**PSDI**	1.52 + 0.49
**BIS**, mean (SD)	
**A**	9.45 + 2.21
**M**	12.44 + 3.48
**SC**	13.17 + 3.16
**CC**	12.14 + 2.56
**P**	7.48 + 1.94
**CI**	5.42 + 1.59
**AI**	14.86 + 2.86
**MI**	19.92 + 4.39
**NPI**	25.30 + 4.86
**Total**	60.08 + 9.81
**TEMPS**, mean (SD)	
**DYS**	9.74 + 4.48
**CYCL**	8.39 + 5.76
**HYPER**	10.88 + 3.95
**IRR**	7.14 + 6.37
**ANX**	10.75 + 7.58
**HIV Stigma Scale**, mean (SD)	
**PS**	36.01 + 12.71
**DC**	28.22 + 6.27
**NSI**	26.30 + 7.77
**CwPA**	45.41 + 12.61
**Total**	91.05 + 22.83

**Table 2:** Results of moderation analyses examining whether affective temperament traits moderate the relationship between depressive symptoms and perceived HIV-related stigma. Values reported include unstandardized regression coefficients (B), standard errors (SE), standardized coefficients (β), t-values, *p*-values, and 95% confidence intervals. * Significant at *p* < 0.05; ** significant after Bonferroni correction (adjusted α = 0.025). Note: HAMD (Hamilton Depression Rating Scale) TEMPS-ANX (anxiety/anxious); TEMPS-CYCL (cyclothymic); TEMPS-DYS (depressive); TEMPS-HYPER (hyperthymic); IRR (irritable). Background shading highlights statistically significant correlations (*p* < 0.05).

Predictor Variable	B	S.E.	β	t	*p*-Value	95% CI (Lower–Upper)
**Model 1: Negative self-image**						
TEMPS-DYS	0.449	0.327	0.262	1.374	0.173	−0.202–1.100
TEMPS-CYCL	0.396	0.266	0.298	1.488	0.141	−0.134–0.927
TEMPS-HYPER	−0.338	0.195	−0.172	−1.731	0.087	−0.726–0.051
TEMPS-IRR	−0.027	0.220	−0.023	−0.125	0.901	−0.466–0.411
TEMPS-ANX	−0.752	0.239	−0.733	−3.147	0.002	−1.227–−0.276
HAMD	0.719	0.153	0.533	4.687	0.000	0.414–1.024
TEMPS-DYS × HAMD	−0.079	0.053	−0.295	−1.498	0.138	−0.185–0.026
TEMPS-CYCL × HAMD	−0.027	0.043	−0.114	−0.638	0.525	−0.113–0.058
TEMPS-HYPER × HAMD *	0.088	0.039	0.247	2.258	0.027	0.010–0.166
TEMPS-IRR × HAMD	−0.042	0.040	−0.165	−1.047	0.298	−0.122–0.038
TEMPS-ANX × HAMD **	0.125	0.035	0.748	3.588	0.001	0.056–0.194
**Model 2: Total stigma score**						
TEMPS-DYS	0.931	0.998	0.186	0.933	0.354	−1.056–2.919
TEMPS-CYCL	1.160	0.814	0.298	1.425	0.158	−0.460–2.780
TEMPS-HYPER	−0.766	0.596	−0.133	−1.286	0.202	−1.953–0.420
TEMPS-IRR	−0.665	0.672	−0.188	−0.989	0.326	−2.003–0.674
TEMPS-ANX	−1.533	0.730	−0.511	−2.100	0.039	−2.986–−0.080
HAMD	1.912	0.469	0.485	4.080	0.000	0.979–2.845
TEMPS-DYS × HAMD	−0.219	0.162	−0.278	−1.349	0.181	−0.541–0.104
TEMPS-CYCL × HAMD	−0.080	0.131	−0.113	−0.607	0.546	−0.341–0.182
TEMPS-HYPER × HAMD	0.222	0.120	0.213	1.859	0.067	−0.016–0.461
TEMPS-IRR × HAMD	−0.102	0.123	−0.137	−0.830	0.409	−0.347–0.143
TEMPS-ANX × HAMD **	0.336	0.106	0.687	3.156	0.002	0.124–0.547

## Data Availability

The data presented in this study are available from the corresponding author on reasonable request. Due to the sensitive nature of the clinical and psychiatric information, restrictions apply to the availability of these data in order to protect participant confidentiality.

## Supplementary Materials

The following supporting information can be downloaded at: https://www.mdpi.com/article/10.3390/brainsci15080786/s1, Table S1: Univariate regression analyses examining the associations between psychological, clinical, and sociodemographic variables and each dimension of HIV-related stigma; Table S2: Results of multiple multivariate linear regression models examining the predictors of stigma-related outcomes.

## Abbreviations

The following abbreviations are used in this manuscript:

**BIS-11**	Barratt Impulsiveness Scale
**BPRS**	Brief Psychiatric Rating Scale
**HAMA**	Hamilton Anxiety Rating Scale
**HAMD**	Hamilton Depression Rating Scale
**HIV**	Human Immunodeficiency Virus
**KMDRS**	Koukopoulos Mixed Depression Rating Scale
**MLR**	Multivariate linear regression
**MMSE**	Mini-Mental State Examination
**PLWH**	People living with HIV
**SCID-5-CV**	Structured Clinical Interview for DSM-5, Clinical Version
**SCID-5-PD**	Structured Clinical Interview for DSM-5, Personality Disorders
**SCL-90-R**	Symptom Checklist-90 Revised
**TEMPS-A**	Temperament Evaluation of Memphis, Pisa, Paris and San Diego
**YMRS**	Young Mania Rating Scale
