# Supplementary material for: Anxious Traits Intensify the Impact of Depressive Symptoms on Stigma in People Living with HIV
Source: Brain Sci. 2025 Jul 24;15(8):786. doi: 10.3390/brainsci15080786 (PMC12384492; doi:10.3390/brainsci15080786)
Supplement: Supplementary file 1 [file brainsci-15-00786-s001.zip › brainsci-3726883-supplementary.pdf]

Supplementary table S1. Univariate regression analyses examining the associations between psychological, clinical, and sociodemographic variables and each dimension of HIV-related stigma. ***Note*.** ART (Antiretroviral Therapy), BIS A (Barratt Impulsiveness Scale – Attentional Impulsiveness), BIS AI (Attentional Instability), BIS CC (Cognitive Complexity), BIS CI (Cognitive Instability), BIS M (Motor Impulsiveness), BIS MI (Motor Impulsiveness Index), BIS NPI (Non-Planning Impulsiveness), BIS P (Perseverance), BIS SC (Self-Control), BIS Total (Total score of the Barratt Impulsiveness Scale-11), BPRS (Brief Psychiatric Rating Scale), CD4+ (Cluster of Differentiation 4 cell count, cells/mm^3), HAMD (Hamilton Depression Rating Scale), HAMA (Hamilton Anxiety Rating Scale), KMDRS (Kutcher Modified Depression Rating Scale), SCL90-R ANX (Symptom Checklist-90-Revised – Anxiety), SCL90-R DEP (Depression), SCL90-R GSI (Global Severity Index), SCL90-R HOS (Hostility), SCL90-R I-S (Interpersonal Sensitivity), SCL90-R O-C (Obsessive–Compulsive), SCL90-R PAR (Paranoid Ideation), SCL90-R PHOB (Phobic Anxiety), SCL90-R PSY (Psychoticism), SCL90-R PSDI (Positive Symptom Distress Index), SCL90-R PST (Positive Symptom Total), SCL90-R SOM (Somatization), YMRS (Young Mania Rating Scale).

	**Dependent variables**				
**Predictors**	**Personalized Stigma**	**Disclosure Concerns**	**Negative Self-Image**	**Public attitudes about PLWHA**	**Total Stigma Score**
**HAMD**	β = 0.431, *p* < 0.001	β = 0.350, *p* = 0.001	β = 0.506, *p* < 0.001	β = 0.429, *p* < 0.001	β = 0.464, *p* < 0.001
**HAMA**	β = 0.371, *p* < 0.001	β = 0.285, *p* = 0.005	β = 0.418, *p* < 0.001	β = 0.376, *p* < 0.001	β = 0.392, *p* < 0.001
**YMRS**	β = 0.140, *p* = 0.180	β = - 0.063, *p* = 0.546	β = 0.111, *p* = 0.290	β = 0.075, *p* = 0.474	β = 0.075, *p* = 0.476
**KMDRS**	β = 0.227, *p* = 0.030	β = 0.114, *p* = 0.280	β = 0.264, *p* = 0.011	β = 0.212, *p* = 0.043	β = 0.217, *p* = 0.037
**BPRS**	β = 0.328, *p* = 0.001	β = 0.321, *p* = 0.002	β = 0.362, *p* < 0.001	β = 0.375, *p* < 0.001	β = 0.365, *p* < 0.001
**SCL90 SOM**	β = 0.233, *p* = 0.023	β = 0.253, *p* = 0.013	β = 0.324, *p* = 0.001	β = 0.182, *p* = 0.078	β = 0.259, *p* = 0.011
**SCL90 O-C**	β = 0.340, *p* = 0.001	β = 0.298, *p* = 0.003	β = 0.395, *p* < 0.001	β = 0.340, *p* = 0.001	β = 0.355, *p* < 0.001
**SCL90 I-S**	β = 0.398, *p* < 0.001	β = 0.421, *p* < 0.001	β = 0.470, *p* < 0.001	β = 0.409, *p* < 0.001	β = 0.452, *p* < 0.001
**SCL90 DEP**	β = 0.336, *p* = 0.001	β = 0.338, *p* = 0.001	β = 0.412, *p* < 0.001	β = 0.357, *p* < 0.001	β = 0.373, *p* < 0.001
**SCL90 ANX**	β = 0.387, *p* < 0.001	β = 0.352, *p* < 0.001	β = 0.453, *p* < 0.001	β = 0.369, *p* < 0.001	β = 0.408, *p* < 0.001
**SCL90 HOS**	β = 0.305, *p* = 0.003	β = 0.358, *p* < 0.001	β = 0.418, *p* < 0.001	β = 0.353, *p* < 0.001	β = 0.376, *p* < 0.001
**SCL90 PHOB**	β = 0.321, *p* = 0.002	β = 0.254, *p* = 0.013	β = 0.315, *p* = 0.002	β = 0.314, *p* = 0.002	β = 0.330, *p* = 0.001
**SCL90 PAR**	β = 0.283, *p* = 0.005	β = 0.248, *p* = 0.016	β = 0.314, *p* = 0.002	β = 0.288, *p* = 0.005	β = 0.298, *p* = 0.003
**SCL90 PSY**	β = 0.389, *p* < 0.001	β = 0.387, *p* < 0.001	β = 0.475, *p* < 0.001	β = 0.400, *p* < 0.001	β = 0.432, *p* < 0.001
**SCL90 GSI**	β = 0.372, *p* < 0.001	β = 0.358, *p* < 0.001	β = 0.450, *p* < 0.001	β = 0.369, *p* < 0.001	β = 0.404, *p* < 0.001
**SCL90 PST**	β = 0.370, *p* < 0.001	β = 0.363, *p* < 0.001	β = 0.407, *p* < 0.001	β = 0.342, *p* = 0.001	β = 0.388, *p* < 0.001
**SCL90 PSDI**	β = 0.341, *p* = 0.001	β = 0.383, *p* < 0.001	β = 0.461, *p* < 0.001	β = 0.385, *p* < 0.001	β = 0.412, *p* < 0.001
**BIS A**	β = 0.263, *p* = 0.010	β = 0.206, *p* = 0.045	β = 0.231, *p* = 0.025	β = 0.280, *p* = 0.006	β = 0.261, *p* = 0.011
**BIS M**	β = 0.128, *p* = 0.218	β = 0.169, *p* = 0.101	β = 0.090, *p* = 0.388	β = 0.120, *p* = 0.246	β = 0.129, *p* = 0.211
**BIS SC**	β = 0.075, *p* = 0.471	β = 0.066, *p* = 0.524	β = 0.066, *p* = 0.524	β = 0.068, *p* = 0.511	β = 0.065, *p* = 0.534

**BIS CC**	β = 0.139, *p* = 0.179	β = 0.006, *p* = 0.955	β = 0.119, *p* = 0.251	β = 0.119, *p* = 0.249	β = 0.109, *p* = 0.295
**BIS P**	β = 0.048, *p* = 0.647	β = 0.015, *p* = 0.884	β = 0.123, *p* = 0.236	β = 0.019, *p* = 0.857	β = 0.049, *p* = 0.639
**BIS CI**	β = 0.053, *p* = 0.612	β = 0.036, *p* = 0.733	β = 0.045, *p* = 0.668	β = 0.012, *p* = 0.906	β = 0.035, *p* = 0.737
**BIS AI**	β = 0.232, *p* = 0.024	β = 0.178, *p* = 0.084	β = 0.202, *p* = 0.049	β = 0.222, *p* = 0.030	β = 0.220, *p* = 0.032
**BIS MI**	β = 0.123, *p* = 0.234	β = 0.142, *p* = 0.169	β = 0.125, *p* = 0.226	β = 0.105, *p* = 0.313	β = 0.125, *p* = 0.227
**BIS NPI**	β = 0.122, *p* = 0.240	β = 0.046, *p* = 0.656	β = 0.106, *p* = 0.308	β = 0.107, *p* = 0.301	β = 0.099, *p* = 0.339
**BIS Total**	β = 0.183, *p* = 0.075	β = 0.139, *p* = 0.180	β = 0.168, *p* = 0.104	β = 0.165, *p* = 0.110	β = 0.170, *p* = 0.101
**Age**	β = 0.096, *p* = 0.350	β = 0.059, *p* = 0.568	β = 0.072, *p* = 0.487	β = 0.083, *p* = 0.419	β = 0.081, *p* = 0.435
**Gender**	β = 0.079, *p* = 0.446	β = 0.236, *p* = 0.020	β = 0.110, *p* = 0.286	β = 0.134, *p* = 0.193	β = 0.150, *p* = 0.146
**Marital status**	β = - 0.092, *p* = 0.371	β = - 0.087, *p* = 0.399	β = - 0.031, *p* = 0.764	β = - 0.083, *p* = 0.419	β = - 0.098, *p* = 0.341
**Level of education**	β = - 0.127, *p* = 0.219	β = - 0.126, *p* = 0.222	β = - 0.210, *p* = 0.040	β = - 0.092, *p* = 0.373	β = - 0.138, *p* = 0.181
**Employment status**	β = - 0.176, *p* = 0.088	β = - 0.297, *p* = 0.003	β = - 0.270, *p* = 0.008	β = - 0.186, *p* = 0.071	β = - 0.244, *p* = 0.017
**CD4+ levels (cells/mm^3)**	β = - 0.085, *p* = 0.513	β = 0.044, *p* = 0.734	β = - 0.012, *p* = 0.926	β = 0.017, *p* = 0.895	β = - 0.010, *p* = 0.941
**Years from diagnosis**	β = 0.153, *p* = 0.137	β = 0.159, *p* = 0.121	β = 0.179, *p* = 0.080	β = 0.109, *p* = 0.291	β = 0.155, *p* = 0.132
**Years of ART therapy**	β = 0.180, *p* = 0.086	β = 0.142, *p* = 0.175	β = 0.175, *p* = 0.096	β = 0.137, *p* = 0.193	β = 0.170, *p* = 0.105
**Number of ART medications taken**	β = - 0.061, *p* = 0.569	β = - 0.076, *p* = 0.477	β = - 0.122, *p* = 0.256	β = - 0.077, *p* = 0.476	β = - 0.097, *p* = 0.367
**Current psychiatric disorder**	β = 0.073, *p* = 0.480	β = 0.205, *p* = 0.046	β = 0.155, *p* = 0.134	β = 0.148, *p* = 0.153	β = 0.139, *p* = 0.180
**Past psychiatric disorder**	β = 0.129, *p* = 0.214	β = 0.191, *p* = 0.063	β = 0.197, *p* = 0.055	β = 0.145, *p* = 0.160	β = 0.170, *p* = 0.099
**Past psychiatric hospitalization**	β = - 0.058, *p* = 0.576	β = 0.112, *p* = 0.278	β = 0.060, *p* = 0.559	β = 0.001, *p* = 0.992	β = 0.022, *p* = 0.830
**Past suicide attempts**	β = 0.069, *p* = 0.503	β = - 0.052, *p* = 0.616	β = - 0.015, *p* = 0.884	β = 0.019, *p* = 0.858	β = 0.016, *p* = 0.879
**Family history of psichiatric disorders**	β = 0.113, *p* = 0.272	β = 0.110, *p* = 0.288	β = 0.206, *p* = 0.044	β = 0.096, *p* = 0.350	β = 0.142, *p* = 0.168
**Number of psychotropic drugs taken**	β = 0.020, *p* = 0.847	β = - 0.109, *p* = 0.288	β = - 0.049, *p* = 0.637	β = - 0.015, *p* = 0.885	β = - 0.040, *p* = 0.696
**Current substance use**	β = - 0.055, *p* = 0.597	β = - 0.138, *p* = 0.179	β = - 0.044, *p* = 0.673	β = - 0.092, *p* = 0.370	β = - 0.092, *p* = 0.375
**Past substance use**	β = 0.057, *p* = 0.578	β = - 0.015, *p* = 0.884	β = 0.096, *p* = 0.350	β = - 0.021, *p* = 0.841	β = 0.025, *p* = 0.811

Supplementary table S2. Results of multiple multivariate linear regression models examining the predictors of stigma-related outcomes. Each column represents a separate model with a different stigma dimension as the dependent variable. Values reported include unstandardized regression coefficients (B), standard errors (SE), standardized coefficients (β), t-values, p-values, and 95% confidence intervals. *Significant at p < .05; **Significant after Bonferroni correction (adjusted $\alpha = 0.01$). *Note.* , BPRS (Brief Psychiatric Rating Scale), HAMD (Hamilton Depression Rating Scale), HAMA (Hamilton Anxiety Rating Scale), KMDRS (Kutcher Modified Depression Rating Scale), SCL90-R ANX (Symptom Checklist-90-Revised – Anxiety), SCL90-R DEP (Depression), SCL90-R GSI (Global Severity Index), SCL90-R HOS (Hostility), SCL90-R I-S (Interpersonal Sensitivity), SCL90-R O-C (Obsessive–Compulsive), SCL90-R PAR (Paranoid Ideation), SCL90-R PHOB (Phobic Anxiety), SCL90-R PSY (Psychoticism), SCL90-R PSDI (Positive Symptom Distress Index), SCL90-R PST (Positive Symptom Total), SCL90-R SOM (Somatization).

**Predictor variable**	**B**	**S.E.**	**β**	**t**	***p*-value**	**95% CI (Lower–Upper)**
**Model 1: Personalized stigma**						
HAMD	1.003	0.577	0.451	1.738	0.087	0.148 - 2.153
HAMA	−0.049	0.498	−0.021	−0.097	0.923	1.043 - 0.946
KMDRS	0.083	0.560	0.024	0.147	0.883	1.035 - 1.200
BPRS	0.303	0.212	0.185	1.431	0.157	0.119 - 0.725
SCL90 SOM	1.611	8.227	0.082	0.196	0.845	14.800 - 18.023
SCL90 O-C	4.760	7.193	0.300	0.662	0.510	9.590 - 19.109
SCL90 I-S	11.658	6.715	0.617	1.736	0.087	1.739 - 25.055
SCL90 DEP	1.247	9.967	0.079	0.125	0.901	18.636 - 21.131
SCL90 ANX	10.533	7.794	0.503	1.351	0.181	5.016 - 26.083
SCL90 HOS	3.266	6.024	0.157	0.542	0.589	8.752 - 15.284
SCL90 PHOB	3.624	7.129	0.112	0.508	0.613	10.599 - 17.846
SCL90 PAR	2.459	5.527	0.127	0.445	0.658	8.568 - 13.486
SCL90 PSY	8.148	9.031	0.386	0.902	0.370	9.867 - 26.164
SCL90 GSI	-46.584	55.290	−2.162	−0.843	0.402	156.884 - 63.716
SCL90 PST	0.029	0.215	0.049	0.136	0.892	0.399 - 0.458
SCL90 PSDI	-3.885	7.232	−0.153	−0.537	0.593	18.313 - 10.542
BIS A	1.379	1.115	0.248	1.237	0.220	0.846 - 3.604
BIS AI	-0.406	0.958	−0.094	−0.423	0.673	2.318 - 1.506
**Model 2: Disclosure concerns**						
HAMD	0.364	0.254	0.336	1.431	0.157	-0.144 - 0.871
HAMA	-0.202	0.227	-0.179	-0.889	0.377	-0.654 - 0.251
BPRS	0.127	0.098	0.159	1.297	0.199	-0.069 - 0.323

SCL90 SOM	4.990	3.801	0.518	1.313	0.194	-2.596 - 12.575
SCL90 O-C	3.377	3.305	0.436	1.022	0.311	-3.219 - 9.972
SCL90 I-S*	8.055	3.145	0.866	2.562	0.013	1.780 - 14.330
SCL90 DEP	3.284	4.747	0.422	0.692	0.491	-6.188 - 12.756
SCL90 ANX	4.779	3.812	0.467	1.254	0.214	-2.828 - 12.386
SCL90 HOS	5.660	2.837	0.555	1.995	0.050	-0.001 - 11.321
SCL90 PHOB	1.586	3.150	0.100	0.504	0.616	-4.698 - 7.871
SCL90 PAR	-1.103	2.505	-0.116	-0.440	0.661	-6.101 - 3.896
SCL90 PSY	7.134	4.226	0.687	1.688	0.096	-1.300 - 15.567
SCL90 GSI	-42.836	26.418	-4.064	-1.621	0.110	-95.552 - 9.880
SCL90 PST	0.125	0.103	0.433	1.215	0.229	-0.080 - 0.331
SCL90 PSDI	4.664	3.316	0.376	1.407	0.164	-1.953 - 11.282
BIS A	0.029	0.310	0.010	0.092	0.927	-0.590 - 0.647
Gender	2.162	1.291	0.169	1.675	0.099	-0.414 - 4.737
Employment status*	-3.311	1.578	-0.231	-2.099	0.040	-6.459 - -0.163
Current psychiatric disorder	-0.800	1.817	-0.054	-0.440	0.661	-4.426 - 2.827
**Model 3: Negative self-image**						
HAMD**	0.902	0.316	0.659	2.856	0.006	0.271 - 1.533
HAMA	-0.237	0.281	-0.167	-0.845	0.401	-0.798 - 0.324
KMDRS	-0.231	0.313	-0.111	-0.737	0.464	-0.856 - 0.395
BPRS	0.171	0.120	0.169	1.422	0.160	-0.069 - 0.410
SCL90 SOM	3.276	4.566	0.269	0.717	0.476	-5.843 - 12.395
SCL90 O-C	2.802	3.968	0.287	0.706	0.483	-5.122 - 10.727
SCL90 I-S	5.836	3.797	0.502	1.537	0.129	-1.747 - 13.419
SCL90 DEP	0.289	5.611	0.030	0.052	0.959	-10.916 - 11.495
SCL90 ANX	3.402	4.510	0.264	0.754	0.453	-5.606 - 12.410
SCL90 HOS	4.499	3.529	0.351	1.275	0.207	-2.549 - 11.548
SCL90 PHOB	-0.940	3.968	-0.047	-0.237	0.814	-8.865 - 6.985
SCL90 PAR	-0.274	3.112	-0.023	-0.088	0.930	-6.490 - 5.941
SCL90 PSY	6.294	5.068	0.482	1.242	0.219	-3.827 - 16.416
SCL90 GSI	-21.747	31.485	-1.639	-0.691	0.492	-84.627 - 41.134
SCL90 PST	-0.089	0.125	-0.242	-0.707	0.482	-0.339 - 0.162
SCL90 PSDI	-0.367	4.078	-0.023	-0.090	0.929	-8.512 - 7.778
BIS A	0.119	0.619	0.035	0.192	0.848	-1.117 - 1.356
BIS AI	-0.170	0.526	-0.064	-0.324	0.747	-1.220 - 0.880
Level of education	-2.161	1.140	-0.178	-1.894	0.063	-4.438 - 0.117

Employment status*	-3.931	1.949	-0.218	-2.017	0.044	-7.822 - -0.039
Family history of psichiatric disorders	1.839	1.750	0.108	1.050	0.297	-1.657 - 5.334
**Model 4: Public attitudes about PLWHA**						
HAMD*	1.088	0.524	0.493	2.077	0.041	0.043 - 2.132
HAMA	-0.004	0.461	-0.002	-0.009	0.992	-0.923 - 0.914
KMDRS	-0.208	0.526	-0.062	-0.396	0.693	-1.256 - 0.840
BPRS	0.396	0.199	0.243	1.992	0.050	0.000 - 0.792
SCL90 O-C	4.666	4.197	0.296	1.112	0.270	-3.705 - 13.038
SCL90 I-S*	11.566	4.464	0.617	2.591	0.012	2.664 - 20.469
SCL90 DEP	3.255	5.272	0.206	0.617	0.539	-7.260 - 13.770
SCL90 ANX	8.342	5.879	0.401	1.419	0.160	-3.383 - 20.068
SCL90 HOS	6.060	4.185	0.293	1.448	0.152	-2.287 - 14.407
SCL90 PHOB	3.743	5.875	0.117	0.637	0.526	-7.975 - 15.460
SCL90 PAR	2.544	4.576	0.133	0.556	0.580	-6.582 - 11.670
SCL90 PSY	8.354	5.802	0.398	1.440	0.154	-3.218 - 19.926
SCL90 GSI*	-51.107	23.430	-2.390	-2.181	0.033	-97.837 - -4.377
SCL90 PST	0.034	0.201	0.058	0.172	0.864	-0.366 - 0.435
SCL90 PSDI	-0.389	6.769	-0.015	-0.057	0.954	-13.890 - 13.113
BIS A	1.278	1.047	0.231	1.221	0.226	-0.810 - 3.365
BIS AI	-0.383	0.898	-0.089	-1.426	0.671	-2.174 - 1.408
**Model 5: Total stigma score**						
HAMD**	2.603	0.946	0.657	2.751	0.008	0.715 - 4.491
HAMA	-0.647	0.838	-0.157	-0.771	0.443	-2.319 - 1.026
KMDRS	-0.532	0.928	-0.088	-0.573	0.569	-2.385 - 1.321
BPRS	0.693	0.355	0.237	1.953	0.055	-0.015 - 1.402
SCL90 SOM	12.226	13.647	0.347	0.896	0.374	-15.014 - 39.466
SCL90 O-C	13.292	11.885	0.470	1.118	0.267	-10.431 - 37.015
SCL90 I-S*	27.537	11.284	0.817	2.440	0.017	5.014 - 50.059
SCL90 DEP	8.353	16.743	0.295	0.499	0.619	-25.066 - 41.773
SCL90 ANX	15.939	13.526	0.427	1.178	0.243	-11.059 - 42.937
SCL90 HOS	16.807	10.581	0.453	1.588	0.117	-4.313 - 37.927
SCL90 PHOB	6.955	11.796	0.121	0.590	0.557	-16.590 - 30.500
SCL90 PAR	3.126	9.084	0.091	0.344	0.732	-15.007 - 21.258
SCL90 PSY	18.602	15.174	0.492	1.226	0.225	-11.686 - 48.890

SCL90 GSI	-127.622	94.379	-3.320	1.352	0.181	-316.003 - 60.759
SCL90 PST	0.044	0.373	0.042	0.118	0.906	-0.701 - 0.789
SCL90 PSDI	3.359	12.125	0.074	0.277	0.783	-20.842 - 27.560
BIS A	1.535	1.836	0.155	0.836	0.406	-2.129 - 5.199
BIS AI	-0.592	1.576	-0.077	-0.376	0.708	-3.738 - 2.554
Employment status	-8.917	5.821	-0.171	-1.532	0.130	-20.535 - 2.701
